# Epidemiology of High Fertility Status among Women of Reproductive Age in Wonago District, Gedeo Zone, Southern Ethiopia: A Community-Based Cross-Sectional Study

**DOI:** 10.1155/2020/2915628

**Published:** 2020-05-21

**Authors:** Medhin Girmay Reda, Girma Tenkolu Bune, Mohammed Feyisso Shaka

**Affiliations:** ^1^College of Medicine and Health Sciences, Adigrat University, Adigrat, Ethiopia; ^2^School of Public Health, College of Health Sciences and Medicine, Dilla University, Dilla, Ethiopia

## Abstract

**Background:**

High fertility remains one of the most important public health issues hampering the health and welfare of mothers and the survival of their children in developing nations. In Ethiopia, the high fertility rate has been seen for a long historical period with some pocket areas of high fertility still showing poor improvement. Hence, this study was aimed at determining the magnitude of high fertility status (number of children ever born alive ≥ 5) and associated factors among women of the reproductive age group in Wonago district.

**Methods:**

A community-based cross-sectional study was conducted on randomly selected 512 women in Wonago district. Data were collected using a pretested structured interviewer administered questionnaire. Data was entered into EpiData version 3.1 and then analyzed by SPSS version 25. Logistic regression was used to analyze the data, and the adjusted odds ratio with the 95% confidence interval was computed, and a significant association was declared at *p* value ≤ 0.05.

**Result:**

This study revealed that 354 (69.1%) of the respondents have high fertility. High fertility is independently associated with residing in rural area [AOR = 4.88, 95% CI: 3.21, 7.86], desire for children [AOR = 6.97, 95% CI: 3.24, 11.40], history of under-five child mortality [AOR =5.32, 95% CI: 2.59, 8.43], poor knowledge of contraception [AOR = 2.67, 95% CI: 1.66, 4.04], and low wealth tertile [AOR = 2.21, 95% CI: 1.51, 3.58]. On the other hand, women with age at first birth above 18 years [AOR = 0.34, 95% CI: 0.17, 0.68] and those with birth interval ≥ 24 months [AOR = 0, 26, 95% CI: 0.14, 0.49] were less likely to have high fertility. *Conclusion and Recommendation*. The substantial number of women in the study area has high fertility status far away from the country's costed implementation plan of reducing the total fertility rate to 3.0. Considering these, much is needed to be done among poor, rural residents, who have not yet attained their desired number of children, and on enhancing the knowledge of mothers towards contraceptive methods.

## 1. Introduction

Based on the demographic concept, fertility is the product or output of reproduction and not the ability to have children. This is to mean that fertility is the actual reproductive performance of a couple [1]. The population's fertility is measured by children ever born, and it shows how many children a certain age cohort of women who have completed childbearing actually produced during their childbearing years [2]. It is one of the three principal components of population dynamics that determines the size and structure of the population of a country [3]. High fertility is defined as five or more births per woman over the reproductive period. It affects health of children and their mothers, detracts from human capital investment, slows economic growth, and exacerbates environmental threats [4].

World population has increased by 2 billion people over the past 25 years, from 5.3 billion in 1990 to 7.3 billion in 2015, and globally, the total fertility rate is 2.5 children per woman Africa has the highest rate of population growth among the main areas of high fertility where the population growth rate is growing at 2.55% annually in 2015 [5].

The United Nations (UN) has projected the median world population for the year 2100 to be 11 billion, and the median projected for Mid-African Countries (MAC) is more than 3.97 billion [6, 7]. High and slowly decreasing fertility resulted in the remarkable increase in the projected population of the great majority of the sub-Saharan (and hence Mid-African Countries) African countries [8]. The fertility decline in this region started nearly 20 years later compared to the rest of the developing countries [5] and, once begun, is estimated to have been one-fourth as fast as in Asia and Latin America at the equivalent demographic stage [9].

Uncontrolled fertility has adverse influences on socioeconomic, demographic, and environmental development of a country [10]. Women with five or more pregnancies have a significantly higher risk of dying due to maternal causes, and there is also poor child survival for women from countries with high fertility [4]. For instance, for every 100,000 births in African countries with high fertility, 640 women die due to related complications of pregnancy and childbirth [11].

Sub-Saharan African countries particularly have population growth rates that are outpacing their economic growth. Fertility still remains far above the replacement level in a significant number of countries. Each African mother is replacing herself with nearly two daughters with replacement level fertility of 2.1, which leads to fast population growth [12].

Though there is a significant decrement in fertility in the world since the previous decades, it is still unacceptably high in most of the Sub-Saharan African countries. Despite the substantial decline in the total fertility rate in Ethiopia, the country is still characterized by high fertility and very young population structure. In addition, the pattern of decline in fertility is not evenly shared among different societal characteristics and geographical areas [13]. There is a fall in fertility in some regions of the country while some are showing no change and others still have shown an increment in the total fertility rate [14].

In Ethiopia, the high and persistent fertility rate has been seen for a long period. The TFR has slightly declined from 5.5 in 2000 to 5.4 children per woman in 2005 to 4.8 children per woman in 2011 and to 4.6 children per woman in 2016. Even though a declining trend is shown, like many African countries, the fertility rate of Ethiopia is still high as compared to developed nations [15–17].

In Ethiopia, the levels of maternal mortality and morbidity are also among the highest in the world. Unspaced birth with low maternal health service access seriously affects the health of mothers and children [18, 19]. Recent estimates showed that the country still experiences higher rates of maternal mortality, under-five mortality, and infant mortality of 412 deaths/100,000, 67 deaths/1000 live births, and 48 deaths/1000 live births, respectively [19].

From the Ethiopian population, about 83.5% of people live in rural areas, whereas the high fertility and rapid population growth rate especially in the rural area is unacceptably high with the total fertility rate of above 6 children per woman. Having many children is still considered to be a prestige and gift of God in most Ethiopian rural communities [20]. Particularly, Wonago woreda which is the study area for this study has 864 persons/sq. km in 1999, and currently, the carrying capacity of the land has reached a climax point where about 1300 persons live per square kilometers [21].

Taking into consideration these realities and scenarios, this study was aimed at visualizing the current fertility status and factors contributing to uncontrolled high fertility in the study area. This is important to forward the implication of current activities of the government for the achievement of the desired fertility status, particularly the achievement of the costed implementation plan of reducing total fertility of Ethiopian women to 3.0. Moreover, this study will bring about the where and how to focus on such pocket areas of high fertility.

## 2. Methodology

### 2.1. Study Design, Setting, and Study Period

A community-based cross-sectional study was conducted among women of reproductive age residing in Wonago district, Gedeo zone, Southern Ethiopia, located 377 km to the south of Addis Ababa. The district is well known for being the most densely populated area in the country and having the highest total fertility rate. The district has 21 kebeles (the smallest local administrative classification in Ethiopia), of which 17 kebeles are rural administration and 4 are semiurban. The dominant ethnic group in the study area is the Gedeo ethnic group. This study was conducted from 05 December 2017 to 18 January 2018.

### 2.2. Population

The study population was all women of the reproductive age group from 25 to 49 years. This group of women was taken for this particular study by considering the fact that women in the study area who have given birth at an early age have the possibility of delivering four and above children before they celebrate their twenty-fifth birthday. In this study, women with the number of children ever born alive greater than four were considered having high fertility.

### 2.3. Sample Size and Sampling Technique

The sample size of this study was calculated using the formula for estimation of single population proportion based on the proportion of women with a high fertility rate (*p*) 28% [22], 95% level of confidence with *Z*_*α*/2_ of 1.96, and margin of error (*d*) of 0.05. With this, the calculated sample size was

(1) $$\begin{array}{c} n=\frac{{\left(Z_{\alpha /2}\right)}^{2}p\;\left(1-p\right)}{d^{2}}=\frac{{\left(1.96\right)}^{2}\times 0.28\;\left(1-0.28\right)}{{\left(0.05\right)}^{2}}=\frac{0.7745}{0\;.0025\;}=309.7\approx 310. \end{array}$$

Considering the design effect (DEFF) of 1.5, the sample size becomes 310∗1.5 = 465. Finally, by adding the 10% nonresponse rate to increase the precision by reducing the sampling error, the final sample size was 512.

To select the study participants, the study area was stratified into semiurban and rural, after which two [2] kebeles from semiurban and five [5] kebeles from rural were selected by simple random sampling technique. In each selected kebele, a list of women of reproductive age in the selected age range (25-49 years) and their full addresses was generated by conducting census. From this sampling frame, the final study units were selected from the list of the sampling frame based on probability proportional to size allocation using simple random sampling technique (SRS).

### 2.4. Data Collection Procedure and Data Analysis

A validated structured tool adapted from the survey tools developed by EDHS Survey [19] and World Fertility Survey [23] was used. The tool was also modified after reviewing different published literatures in English version, and a pretest was conducted to customize the objectives of the study and local contexts.

The questionnaire prepared originally in English was then translated to Amharic and the local language (Gedeo-uffa). To check for consistency of the meaning of each question, the questionnaire was further back translated from the local language to Amharic and English by different translators. The data was collected by trained diploma-holder health professionals who are fluent in Gedeo-uffa (local language) and Amharic. Before data collection, the data collectors were trained thoroughly. The principal investigator strictly supervised the overall activities of data collection.

For data analysis, the data was entered into the EpiData version 3.1 statistical software package and later exported into SPSS version 25 [24]. Descriptive analysis was conducted, and logistic regression was done to assess the determinants of high fertility. As a rule of thumb, variables with *p* value less than 0.25 on bivariate analysis were further included in the multivariable analysis model. The adjusted odds ratio with their corresponding confidence interval was used as a measure of association, and *p* values < 0.05 were taken as statistically significant.

The model goodness of fit was tested by the Hosmer and Lemeshow test. Multicollinearity was checked at the cutoff point of VIF > 10 to test if the independent variables were related.

### 2.5. Measurements

#### 2.5.1. Fertility Status

The outcome variable was fertility status measured by the number of children ever born (CEB) alive. It is categorized as low fertility when CEB alive is <5 and high fertility when CEB alive is ≥5. The cutoff point of 5 is taken because the medical and obstetric risk for mothers with the number of CEB greater or equal to 5 is significantly higher compared with that of less than five [25]. It is also based on the population policy of Ethiopia which is aimed at achieving less than five children per woman by the end of 2015.

#### 2.5.2. Children Ever Born (CEB)

CEB represents the number of all children ever born alive to a particular woman in her lifetime fertility experience by the time of the survey.

#### 2.5.3. Knowledge about Contraception

Maternal knowledge towards contraception was assessed by asking six knowledge-related questions. A score of 1 was given if the mother answered the given questions correctly and 0 if not. The sum of each question was computed. Taking into account the distribution of the sum score which was normally distributed, the mean was used to categorize the overall score of each participant into two groups: greater than the mean as good knowledge and less than or equal to the mean as poor knowledge.

#### 2.5.4. Estimation of the Household Wealth Index

Household wealth status was estimated by principal component analysis based on fourteen household variables (source of food, presence of own farmland, type of toilet facility, presence of kitchen, electricity, radio/tape, motorcycle, cotton/sponge/spring mattress, roof of house with corrugated iron sheet, wall type of household, sleeping bed, presence of own animals, and number of cows, oxen, horses/mules/donkeys, goats/sheep, beehives, and hens) that measure household assets. SPSS version 25 software was used to perform principal component analysis (PCA). Household wealth status was estimated with 3 components that explained a total variance of 62.5% and measure of sampling adequacy of 0.72. Finally, wealth status can be categorized into three groups and ranked as low, middle, and high as it was given by the factor analysis result.

### 2.6. Ethical Consideration

Ethical clearance was obtained from Dilla University College of Health Sciences and Medicine Institutional Review Board. Verbal consent was obtained from each study participant after detailed explanation of the main purpose of the study.

## 3. Result

### 3.1. Sociodemographic and Socioeconomic Characteristics of the Respondents

Wide ranges of variables which can potentially affect fertility status were included in the analysis. The mean age of mothers was 35 years (SD = 5.7), and one-third of the mothers (168, 32.8%) were within the age range of 35-39 years. Majority of the study participants are Gedeo in ethnicity (405, 79.1%). Most of the children's parents (433, 84.5%) were married and live together. The residence of 383 (74.8%) mothers is rural setting, and more than half of the mothers (311, 60.7%) were illiterate. Occupationally, 268 (52.5%) mothers were housewives. About 210 (41%) of the participants in the study area were in the lowest wealth tertile (Table 1).

### 3.2. Reproductive Characteristics of the Respondents

Three hundred fifty-four (69.1%) of mothers were found to have “high fertility.” About 17.8% of the participants' husbands in the study area have two or more wives. One hundred fifty-six (30.5%) of the women in the study area have ever aborted or had still birth, and 186 (36.3%) had history of under-five child mortality. The mean number of children ever born (CEB) alive for women (age 25-49 years) in this study is 6.00 with (SD = 2.1), and the parity ranges from 1 to 12. The mean age at first childbirth for the participated women is 17 years with (SD = 1.9) (Table 2).

### 3.3. Mother's Knowledge about Contraceptive Methods

Interviewers collected information regarding knowledge of contraceptive methods, and the result revealed that 397 (77.5%) of women have commendable knowledge about contraceptive methods and 115 (22.5%) of women did have poor knowledge of contraceptive methods.

### 3.4. Factors Associated with High Fertility Status of Participants

After controlling for the effects of possible confounders, current age category, place of residence, mother's education, wealth index, age at first birth, birth interval, knowledge of contraceptive, desire for children, and history of under-five mortality were significantly associated with fertility status (Table 3). As it is literally expected, women in the older age group were more likely to have more children compared to younger women.

Regarding the place of residence, women living in rural areas were about 5 times (AOR = 4.88, 95% CI: 3.21, 7.86) more likely to have high fertility as compared to those living in semiurban settings. Women who have given their first birth at 18 years and above are 65.9% (AOR = 0.34, 95% CI: 0.17, 0.68) more likely to have high fertility compared to women who have given their first birth before 18 years. Similarly, women with average birth spacing ≥ 2 years were about 74% (AOR = 0.26, 95% CI: 0.14, 0.49) less likely to have high fertility compared to those who have a shorter birth interval (less than 2 years).

Women who have poor knowledge of contraceptive methods were nearly 3 times (AOR = 2.67, 95% CI: 1.66, 4.04) more likely to have higher fertility as compared to those who have good knowledge. Mothers within the low wealth tertile were also 2 times (AOR = 2.21, 95% CI: 1.51, 3.58) more likely to have higher fertility as compared to those with high wealth tertile. Women who express their desired number of children as God given were nearly 7 times (AOR = 6.97, 95% CI: 3.24, 11.40) more likely to have high fertility than those who desire to have less than five children. On the other hand, women with history of under-five child mortality were 5 times (AOR = 5.32, 95% CI: 2.59, 8.43) more likely to have higher fertility than women who did not have history of under-five child mortality (Table 3).

## 4. Discussion

The intention of this study was to determine the magnitude and factors associated with high fertility among women of the reproductive age group aged 25-49 years. The finding revealed that 69% (95% CI: 65%-73%) of the 512 respondents had high fertility. This figure is also significantly higher compared to other findings from studies conducted in different parts of Ethiopia, which include a study from Enderta district (51%) [26] and Butajira district (28%) [22]. The main underlying reason for this variation may be the lower mean age at first birth in this study which is smaller than the other observed studies.

Different sociodemographic characteristics have been indicated to be associated with high fertility in these studies. Regarding the household wealth tertile, women within the lower wealth tertile were more likely to have high fertility than those within the high wealth tertile. This could be due to poor access to media and health care services including family planning and low educational status by the poor. This finding is also similar to the facts in EDHS 2016 [19] and findings from Gilgel Gibe Field Research Center of Jimma University [10], in which fertility status has a strong association with wealth quintiles.

Residency also showed a strong association to high fertility in the current study. The fertility levels in the urban and rural areas tend to be different; women who live in the rural area have more children as compared to those living in the urban area. It is generally true that women residing in the urban area stay longer in school, thereby delaying the time for marital engagement. Moreover, the demand for human power in agrarian living condition and the notion of considering the family with a large number of children as a blessed family in the rural area in the district may drive couples to have an increased number of children. This is consistent with previous studies done in the case of Butajira [27], Ghana [28], and Nepal [29]. In a similar context, high fertility was significantly higher among women whose means of subsistence is farming than those living in urban areas that are mainly characterized by trade, access to media, general health knowledge, and better health service information.

Early childbearing is a leading sociodemographic characteristic that indicates the exposure of women to teenage pregnancy. In the area of the current study, early marriage is found to be a common phenomenon as also suggested by the result showing that more than half of the women that participated in this study had their first birth before 18 years of age. Women who gave their first birth at the age of 18 year and above were more likely to have fewer children than those who gave their first birth at an earlier age. For women with their first pregnancy at an earlier age, the period of fertility is obviously longer, resulting in higher fertility. This phenomenon is particularly serious in this study area where a large number of the women have poor knowledge of contraceptive methods. This finding is similar with previous studies done in Kersa district [30], Enderta district [26], Nigeria [31], Nambia [32], and Ghana [28]. However, the issue of early childbearing is considerably alarming in the current study area than the findings from those areas.

Another interesting factor in this study was a desire for compensation or replacement for loss of under-five children. In this study, women who have history of under-five mortality have higher probability of having a large number of children. This finding is consistent with previous studies done in Kersa district [30], Enderta district [26], Gilgel Gibe Field Research Center of Jimma University [10], Butajira [27], Nigeria [33], Nepal [29], and Kenya [34]. This can be explained by the fact that death of the child may create a fear of loss of more children to women resulting in having an unintended higher number of children.

Knowledge of contraception is found to be another determinant factor where women with poor knowledge on contraception were at higher risk of having higher fertility as compared to those who have good knowledge. Similar studies have been found in other parts of the country including Addis Ababa [35], Bale Zone, and South East Ethiopia [36]. As this result indicates, one of the main reasons for higher fertility in the study area may be emanated from the poor knowledge of contraceptive methods which implies that the women could also have a higher unmet need for family planning.

Women of the reproductive age group having an average birth interval of less than 2 years were at higher risk of having high fertility as compared to those with a birth interval more than or equal to two years. This finding is also consistent with a study conducted in Arbaminch district [37] and in Dodota woreda, Arsi zone [38].

This study considered a wide range of fertility variables to see high fertility differentials among women in the study, and it used a reasonable sample of respondents selected with probable sampling technique. However, there are some important limitations in using the finding of this study. As the study is quantitative in nature, there are some hidden factors that need to be addressed by qualitative study. Though the assessment was carefully done to reduce the recall bias, estimation of some sociodemographic variables like age at first birth might have been mistaken.

## 5. Conclusion and Recommendation

The finding of this study implies that fertility status in the study area was alarmingly high, contesting the effort of the country to achieve the lower total fertility rate as planned on the costed implementation plan of 2020. Moreover, the finding implies that the women in the study area have a substantial unmet need for family planning that needs public health attention. Among the impressive factors influencing fertility status were the following: loss of under-five children, low wealth tertile, residing in rural areas, age at first birth, desire for children, and poor knowledge of contraception. Targeted works on knowledge about contraception, the negative effect of early childbearing/marriage, and correcting the desire for children among women in the study area are urgent public health concerns needing due consideration.

## Figures and Tables

**Table 1:** Sociodemographic characteristics of women of reproductive age (25-49 years) in Wonago district, Southern Ethiopia, January 2018 (*n* = 512).

Variables	Variable level	No. (%)
Current age category in years	25-29	88 (17.2%)
	30-34	75 (14.6%)
	35-39	168 (32.8%)
	40-44	111 (21.7%)
	45-49	70 (13.7%)
	Mean ± SD	35 ± 5.7

Residency	Urban	129 (25.2%)
	Rural	383 (74.8%)

Ethnicity	Gedeo	405 (79.1%)
	Oromo	65 (12.7%)
	Silte	16 (3.1%)
	Amhara	26 (5.1%)

Marital status	Married/living together	433 (84.5%)
	Divorced	50 (9.8%)
	Widowed	29 (5.7%)

Mother's educational status	Illiterate	311 (60.7%)
	Primary education	99 (19.3%)
	Secondary & above education	102 (19.9%)

Father's educational level	Illiterate	113 (22.1%)
	Primary education	197 (38.5%)
	Secondary & above education	202 (39.5%)

Occupation of mother	Housewife	268 (52.3%)
	Farmer	152 (29.7%)
	Government employee	30 (5.9%)
	Merchant	62 (12.1%)

Wealth index	Low	210 (41.0%)
	Middle	104 (20.3%)
	High	198 (38.7%)

SD: standard deviation.

**Table 2:** Reproductive characteristics of the study participants in Wonago district, Gedeo zone, January 2018 (*n* = 512).

Variables	Variable level	No. (%)
Fertility status	High fertility	354 (69.1%)
	Low fertility	158 (30.9%)

Currently have children	<5	201 (39.3%)
	≥5	311 (60.7%)

Ever aborted or still birth	No	356 (69.5%)
	Yes	156 (30.5)

Under-five child mortality	Yes	186 (36.3%)
	No	326 (63.7%)

Age at marriage	<18 years	286 (55.9%)
	18 and above	226 (44.1%)
	Mean ± SD	16 ± 2.5

Age at first birth	<18	277 (54.1%)
	18 and above	235 (45.9%)
	Mean ± SD	17 ± 1.9

Age at last birth	≤30	202 (39.5%)
	31 and above	310 (60.5%)
	Mean ± SD	32 ± 4.6

Two or more wives	Yes	91 (17.8%)
	No	421 (82.2%)

Average birth interval	<2 years	322 (62.9%)
	≥2 years	190 (37.1%)
	Mean ± SD	2 ± 0.5

Duration of breastfeeding	<12 months	93 (18.2%)
	12-24 months	206 (40.2%)
	>24 months	213 (41.6%)
	Mean ± SD	24 ± 6.7

Delayed sexual contact after delivery	<2 months	270 (52.7%)
	≥2 months	242 (47.3%)

SD: standard deviation.

**Table 3:** A multivariable analysis of factors associated with fertility status in Wonago district, Southern Ethiopia, January 2018.

Exposure variables	High (354) No. (%)	Low (158) No. (%)	Crude OR (95% CI)	Adjusted OR (95% CI)
Current age category				
25-34 years	117 (53.2%)	103 (46.8%)	1.00	1.00
35-44 years	166 (82.2%)	36 (17.8%)	4.06 (2.59-6.35)	2.94 (1.41-6.10)
45 and above	71 (78.9%)	19 (21.1%)	3.29 (1.90-5.83)	3.39 (1.04- 6.05)
Residency				
Rural	75 (19.6%)	308 (80.4%)	7.41 (4.76-11.50)	4.88 (3.21-7.86)
Urban	83 (64.3%)	46 (35.7%)	1.00	1.00
Age at first birth				
<18 years	230 (83.0%)	47 (17.0%)	1.00	1.00
18 and above	124 (52.8%)	111 (47.2%)	0.23 (0.15-0.34)	0.34 (0.17-0.68)
Wealth index				
Low	152 (72.4%)	58 (27.6%)	2.29 (1.18-5.30)	3.62 (1.86-7.06)
Middle	90 (85.7%)	15 (14.3%)	1.97 (2.19-3.28)	3.03 (1.21-7.58)
High	112 (56.9%)	85 (43.1%)	1.00	1.00
Knowledge of contraceptive				
Poor knowledge	98 (85.2%)	17 (14.8%)	3.18 (1.82-5.53)	3.13 (1.31-7.46)
Good knowledge	256 (64.5%)	141 (35.5%)	1.00	1.00
Birth interval				
<2 years	233 (72.4%)	89 (27.6%)	1.00	1.00
≥2 years	121 (63.7%)	69 (36.3%)	0.67 (0.46-0.98)	0.26 (0.14-0.49)
Desire for children/before birth				
<5	38 (40.9%)	55 (59.1%)	1.00	1.00
≥5	58 (59.2%)	40 (40.8%)	2.10 (1.18-3.74)	3.53 (1.60-7.79)
As God given	258 (80.4%)	63 (19.6%)	5.93 (3.61-9.64)	6.97 (3.24-11.40)
Under-five child mortality				
No	183 (56.1%)	143 (43.9%)	1.00	1.00
Yes	171 (91.9%)	15 (8.1%)	8.91 (5.03-11.77)	5.32 (2.59-8.43)

Variables included in the model are as follows: current age, mother's education, residency, wealth index, age at marriage, age at last birth, age at first birth, birth interval, want for more children, desire for children/before birth, ever aborted/still birth, contraceptive use, knowledge of contraception, and history of under-5 mortality.

## Data Availability

The datasets underlying during the current study are available from the corresponding author on reasonable request.

## Abbreviations

BCC: Behavior change communication
CEB: Child ever born
CSA: Central Statistical Agency
EDHS: Ethiopian Demographic and Health Survey
HAD: Health Development Army
HEW: Health extension worker
IEC: Information education communication
MPH/RH: Masters of Public Health/Reproductive Health
OR: Odds ratio
SNNP: Southern Nations Nationalities and Peoples
SPSS: Statistical Package for the Social Sciences
TFR: Total fertility rate.
